# Interaction between TATA-Binding Protein (TBP) and Multiprotein Bridging Factor-1 (MBF1) from the Filamentous Insect Pathogenic Fungus *Beauveria bassiana*

**DOI:** 10.1371/journal.pone.0140538

**Published:** 2015-10-14

**Authors:** Chi Song, Almudena Ortiz-Urquiza, Sheng-Hua Ying, Jin-Xia Zhang, Nemat O. Keyhani

**Affiliations:** 1 Institute of Agricultural Resources and Regional Planning, Chinese Academy of Agricultural Sciences; Key Laboratory of Microbial Resources, Ministry of Agriculture, Beijing 100081, China; 2 Department of Microbiology and Cell Science, Institute of Food and Agricultural Science, University of Florida, Bldg 981, Museum Rd., Gainesville, FL 32611, United States of America; 3 Institute of Microbiology, College of Life Sciences, Zhejiang University, Hangzhou 310058, China; Hans-Knoell-Institute (HKI), GERMANY

## Abstract

TATA-binding protein (TBP) is a ubiquitous component of eukaryotic transcription factors that acts to nucleate assembly and position pre-initiation complexes. Multiprotein bridging factor 1 (MBF1) is thought to interconnect TBP with gene specific transcriptional activators, modulating transcriptional networks in response to specific signal and developmental programs. The insect pathogen, *Beauveria bassiana*, is a cosmopolitan fungus found in most ecosystems where it acts as an important regulator of insect populations and can form intimate associations with certain plants. In order to gain a better understanding of the function of MBF1 in filamentous fungi, its interaction with TBP was demonstrated. The MBF1 and TBP homologs in *B*. *bassiana* were cloned and purified from a heterologous *E*. *coli* expression system. Whereas purified BbTBP was shown to be able to bind oligonucleotide sequences containing the TATA-motif (Kd ≈ 1.3 nM) including sequences derived from the promoters of the *B*. *bassiana* chitinase and protease genes. In contrast, BbMBF1 was unable to bind to these same target sequences. However, the formation of a ternary complex between BbMBF1, BbTBP, and a TATA-containing target DNA sequence was seen in agarose gel electrophoretic mobility shift assays (EMSA). These data indicate that BbMBF1 forms direct interactions with BbTBP, and that the complex is capable of binding to DNA sequences containing TATA-motifs, confirming that BbTBP can link BbMBF1 to target sequences as part of the RNA transcriptional machinery in fungi.

## Introduction

Promoters for the three eukaryotic RNA polymerases contain variable sets of core and regulatory elements that are the targets for the general and regulatory transcription factors (TFs), respectively. The RNA polymerases themselves are incapable of recognizing target promoters, but are recruited by the polymerase specific TFs, i.e. each polymerase interacts with its own set of general and regulatory TFs. TATA-binding protein (TBP) has been considered a universal TF due to the fact that it is a subunit constituent of the various general transcription factors used by each polymerase [1,2]. Thus, even if a promoter target lacks a defined “TATA” sequence, TBP is often present as part of a subunit complex during transcription mediated by any of the three RNA polymerases.

Multiprotein bridging factor (MBF) was first identified as a non-DNA binding factor that participates in transcriptional activation by the nuclear hormone receptor *fushi tarazu* (*ftz*) gene product in the silkworm *Bombyx mori* and in the fruit fly *Drosophila melanogaster* [3,4]. Yeast MBF1 is activated by the transcription factor GCN4 [5], and human MBF1 has been shown to interact with the mammalian homolog of FTZ-F1, namely, Ad4BP/SF-1 [6]. These findings suggest that MBF acts as a link between TBP and various transcription factors and subsequently MBFs have been found ubiquitously distributed in eukaryotes and archaea, but are absent in prokaryotes [7]. Some organisms contain multiple MBF genes that are thought to mediate interactions between different transcription factors, but all MBFs appear to share a core interaction with TBP [8]. Aspects of this protein-protein interaction have been investigated using yeast MBF1 and TBP [9]. These data have revealed the occurrence of compensatory changes in the respective binding interface domains of these proteins, suggested the co-evolution of specific compensatory amino acids, and thus underscoring the importance of the binding interaction in the functions of these proteins. However, MBF1 is not essential in the budding yeast and its role in other eukaryotes in transcription remains unclear.

Despite their potential critical roles in transcription, there are few reports examining TBP and/or MBF1 in filamentous fungi. The solution structure of (the C-terminal domain of) an MBF1 has been derived from the *Trichoderma reesei* homolog [10]; which aside from work on MBF1 in *Beauveria bassiana*, further described below, are, to the best of our knowledge, the only reports dealing with MBFs from filamentous fungi. Similarly, there are surprisingly few examinations of TBP in filamentous fungi. The TBP gene from *Aspergillus nidulans* has been shown to substitute for the yeast TBP *in vivo* [11] and a conditional lethal disruption of TBP in *Penicillium marneffei* was used to show that TBP in this fungus was essential for filamentous growth, but played a less significant role in growth and development during the yeast phase, a finding that suggests unique role for TBP in filamentous fungi [12]. Thus far, however, there have been no biochemical investigations of the interaction of TBP with cognate TATA-sequences and/or of TBP-MBF1 interactions in filamentous fungi.

The insect pathogenic filamentous fungus, *Beauveria bassiana*, has emerged as a new model system with which to examine fungal stress and development and host-pathogen interactions [13]. The fungus is also of significant interest as a microbial biological control agent capable of targeted different insect pests, especially as a more environmentally friendly alternative to chemical pesticides [14,15]. *B*. *bassiana* displays a broad arthropod host range with infection occurring via attachment and penetration of the host cuticle [16–18]. Fungal hyphae grow in a filamentous form to penetrate the host integument, producing free-floating yeast-like cells termed hyphal bodies once the fungus reaches the hemocoel [19,20]. The hyphal bodies are able to evade the insect immune system, a process that parallels several other microbial pathogens [21,22]. Infected hosts typically die 3–10 d post inoculation, after with the fungus sporulates on the host cadaver to produce new dispersal and infectious cells.

A genetic characterization of the single MBF1 homolog found in *B*. *bassiana* has recently been reported [23]. *BbMBF1* was not essential, but *ΔBbMBF1* mutants displayed abnormal hyphal morphogenesis, were deficient in carbon dependent development, and were reduced in virulence. In addition, loss of BbMBF1 resulted in a wide range of increased stress susceptibilities including, increased sensitivity to osmotic, oxidative, and UV-stress. Here, we report on the heterologous expression and purification of BbMBF1 and TBP and their use in demonstrating (a) the binding of BbTBP to TATA-sequences, revealing a K_d_ = 1.3 nM, (b) the interaction between BbTBP and BbMBF1, and (c) the formation of a ternary complex between BbTBP, cognate TATA sequences, and BbMBF1.

## Materials and Methods

### Strains and reagents

*E coli* strain Rosetta DE3 (Promega, Madison WI, USA) was routinely grown in Luria-Bertani broth (LB) and/or on LB-agar (1.5%) plates containing 50 μg/ml kanamycin as indicated. Reagents and chemicals were purchased from Sigma-Aldrich (St. Louis, MO, USA) and/or Thermo Fischer Scientific (Waltham, MA, USA) except as noted. Potassium ferrocyanide trihydrate (K_4_[Fe(CN)_6_] • 3H_2_O) was purchased from EMD chemicals (Billerica, USA). Ni- and Co-NTA agarose was purchased from Gold Biotech (St. Louis, MO, USA). Thrombin was purchased from (Amersham-Pharmacia Biotech).

### Heterologous expression and purification of BbTBP and BbMBF1

#### Nucleic acid manipulations

Primers used for cloning of *B*. *bassiana TBP* and *MBF1* genes are given in Table 1. The open reading frames (ORFs) corresponding to *BbTBP* and *BbMBF1* were amplified by polymerase chain reaction (PCR) using primer pairs PTBP-F/PTBP-R and PMBF1-F/PMBF1-R, respectively. Forward and reverse primers were designed to contain unique *Eco*RI and *Hind*III, respectively. PCR products were digested with *Eco*RI and *Hind*III, and cloned into respective sites in the pET28a (Novagen) expression vector, to produce plasmids pET28a-*BbTBP* and pET28a-*BbMBF1*, resulting in 6xHis C-terminal-tagged fusion proteins containing thrombin cleavage sites between the His-tag and the protein ORF that could be used to remove the His-tag from the protein. The integrity of the plasmid inserts was verified by sequencing (UF-ICBR Sequencing CORE).

**Table 1:** Oligonucleotides used in this study.

Primer	Function	Nucleotide sequence (5’-3’)	Sequence
PTBP-F	*TBP* expression vector cloning	**GGAATTC**GAGGGCATTCAAACCCACCCT	*TBP* ORF
PTBP-R		**CCCAAGCTTTTA**GACCTTGCGGAAATCTT	*TBP* ORF
PMBF1-F	*MBF1* expression vector cloning	**GGAATTC**TCTGACGACTGGGATTCGCAAAC	*MBF1* ORF
PMBF1-R		**CCCAAGCTTTTA**TTTCTTCTTGGCAAATCT	*MBF1* ORF
PCTRLF	EMSA- TATA^0^	CAGTAAAAGCTTGGTAGTATTTCTTCTCTCTTTCAC	TATA-less chitinase promoter
PCTRLR		GTGAAAGAGAGAAGAAATACTACCAAGCTTTTACTG	Chitinase promoter
PPRA-F	EMSA- TATA^P^	GGAGCGGC**ATAT**GTCAAG**TATA**AAGAGCGGAATGTTTTTC	Protease promoter
PPRA-R		GAAAAACATTCCGCTCTTTATACTTGACATATGCCGCTCC	Protease promoter
PCHIT-F	EMSA –TATA^C^	CAGTAAAAGCTTGGTAGTATT**TATA**TCTTCTCTCTTTCAC	Chitinase promoter
PCHIT-R		GTGAAAGAGAGAAGATATAAATACTACCAAGCTTTTACTG	Chitinase promoter
PCHIT-TEX-F	Fluorescent labeled primer—flTATA^C^	5’-TEX_615_- CAGTAAAAGCTTGGTAGTATT**TATA**TCTTCTCTCTTTCAC	Chitinase promoter

#### Protein expression and purification

Expression plasmids were transformed into competent *E*. *coli* Rosetta DE3 cells. For expression and purification, *E*. *coli* transformants bearing either pET28a-*BbTBP* or pET28a-*BbMBF1* were cultured in LB broth containing 50 μg/ml kanamycin at 37°C with aeration to an optical density of OD_600_ = 0.8 at 37°C, before induction with isopropylthiogalactoside (IPTG) as follows; for the *BbTBP* construct, cultures (2x400 ml) were induced using 0.3 mM (final concentration) IPTG and incubated at 18°C, with aeration for an additional 12 h before harvesting; for *BbMBF1*, cultures were induced using 0.5 mM (final) IPTG and incubated at 30°C with aeration for 4 h. Bacterial cells were harvested by centrifugation, 6000xg, 10 min, washing once with starting buffer (SB, 100 mM NaCl, 100 mM HEPES, 10 mM imidazole, pH 7.5), and then resuspended in 1:50 vol of the same buffer before lysis by French Press. Cell debris was removed by centrifugation (10,000xg, 15 min), and the supernatant was loaded onto a pre-equilibrated (SB buffer) Ni- or Co-NTA agarose column (1 ml). After loading, columns were washed with SB buffer (5 ml) and step-wise eluted (2x5 ml each) with SB buffer containing 100, 200, 300, and 500 mM imidazole. Fractions were assessed for purity by SDS-polyacrylamide gel electrophoresis (PAGE) and pooled (100–500 mM imidazole for BbTBP and BbMBF1). Purified BbTBP was dialyzed against TBP Buffer (20 mM Tris-HC1, pH 7.9, 10% glycerol, 20 mM 2-mercaptoethanol, 1 mM phenylmethanesulfonyl fluoride (PMSF), and 200 mM NaC1, 0.01% Nonidet P-40, [24]) and BbMBF1 was dialyzed against Dialysis Buffer (DB, 10 mM HEPES, pH 7.5, 100 mM NaCl). Proteins were concentrated using PEG and BioMax concentrator (Millipore, Billerica, MA) as needed. Protein concentrations were determined using Pierce™ BCA Protein Assay Kit (Thermo Scientific). Purified proteins were aliquoted and stored at -80°C until use.

### Electrophoretic gel mobility shift assay (EMSA)

Four oligonucleotide templates were prepared for EMSA experiments with primer sequences given in Table 1. Templates were prepared as follows; primer pairs in 0.2 x SSC buffer (3 mM sodium citrate, pH 7, 30 mM NaCl) were heated to 95°C in a water bath for 5 min and allowed to anneal by cooling to room temperature for 30 min. Templates derived from primer pairs PCTRL-F/PCTRL-R, PPRA-F/PPRA-R and PCHIT-F/PCHIT-R were annealed using 1:1 ratios, and the template derived from primer pair PCHIT-TEX-F/PCHIT-R was prepared using a 1:5 primer ratio. Templates were designated as TATA^0^, TATA^P^, TATA^C^, and flTATA^C^, respectively, to indicate the absence of a TATA sequences and derivation from the protease or chitinase gene promoter sequences, and whether the template contained a fluorescent (fl) label, respectively. Incubation mixtures for EMSA reactions (20 μl) contained binding buffer (4 μl, 60 mM KCl, 20 mM Tris (pH 7.9), 5.0 mM MgCl2, 10 mM dithiothreitol, 0.2 mg/ml bovine serum albumin, and 10% glycerol), oligonucleotide template and purified BbTBP and/or BbMBF1 as indicated in the results. Samples were incubated for 30 min at 37°C, analyzed by 6% acrylamide-bisacrylamide non-denaturing gels [25]. In the case of non-labeled templates, gels were stained using the EMSA dual protein-nucleic acid staining kit (Molecular Probes) and visualized using a Gel Doc EZ System (Bio-Rad, Hercules, CA, USA). Experiments using the TEX-615 labeled template were visualized using the same system directly. Band intensities were quantified using the Gel Doc visualization software. All experiments were performed in triplicate and data were analyzed by Excel and GraphPad Prism 5. Protein pI values were calculated using the DNA MAN software (Lynnan Corp, Pointe-Claire, QC, Canada)

## Results

### Expression and purification of BbTBP and MBF1

**Fig 1:**
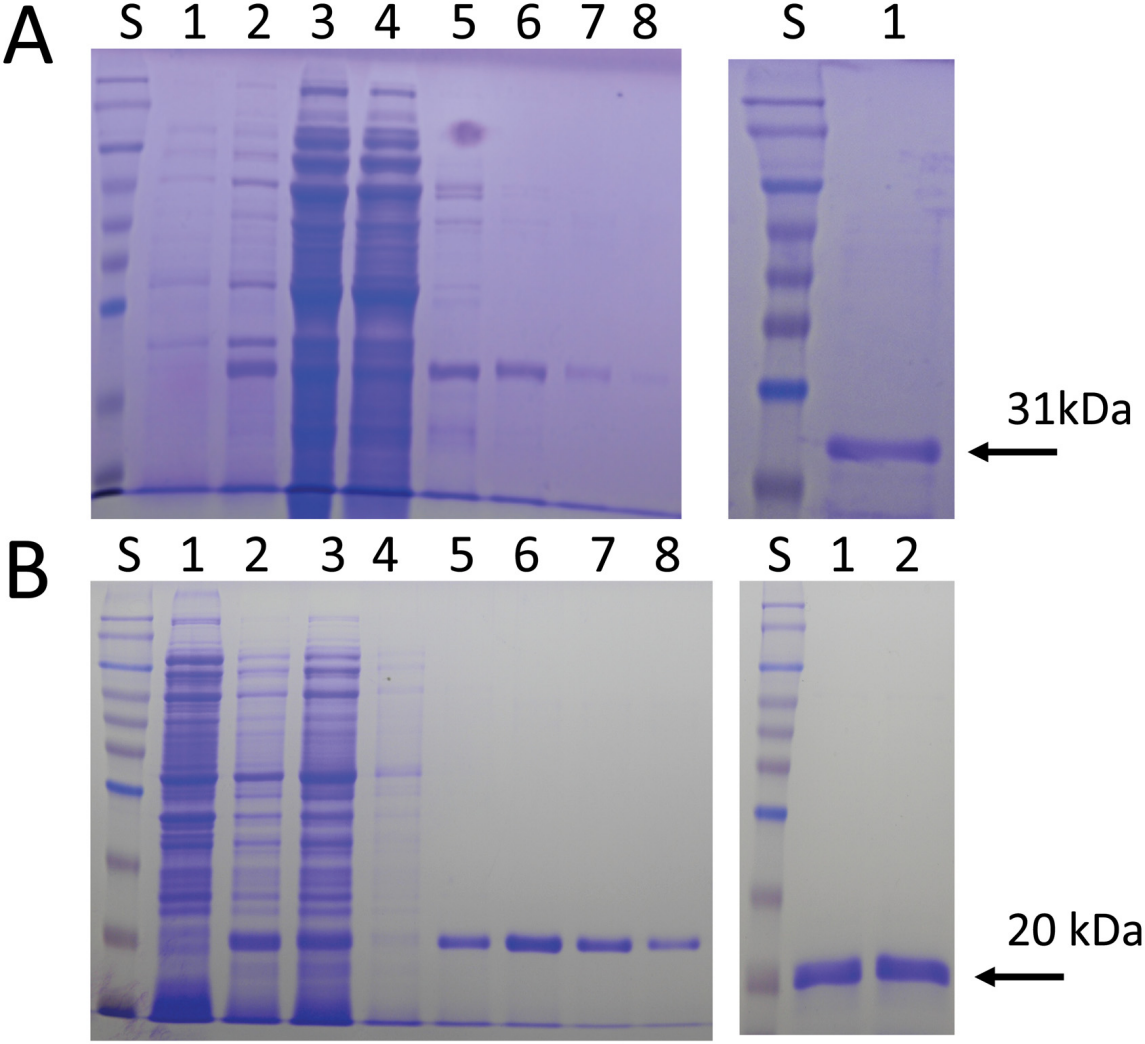
Expression and purification of BbTBP and BbMBF1. (A) SDS-PAGE analysis of BbTBP purification, left panel, lane S, molecular weight markers, lane 1 before induce, lane 2 after induce with 0.3 mM IPTG, 18°C, 12 h, lane 3 flow through, lane 4 wash with starting buffer, lane 5–8 wash with (100, 200, 400, 500) imidazole; right panel, lane 1 final purified, dialyzed product. (B) SDS-PAGE analysis of BbMBF1 purification, left panel, lane S, molecular weight markers, lane 1 before induce, lane 2 after induce with 0.5 mM IPTG, 30°C, 4 h, lane 3 flow through, lane 4 wash with starting buffer, lane 5–8 wash with (100, 200, 400, 500) imidazole; right panel, lane 1 final purified, dialyzed product. Lane 2 final his-tag cleaved product.

The gene loci for *BbTBP* and *BbMBF1* were identified in the *B*. *bassiana* genome sequence database as locus tags: BBA_05384 and BBA_01830, respectively [26]. *BbTBP* and *MBF1* encoded for proteins consisting of 253 amino acids with a pI of 13.7, and 153 amino acid and a pI of 10.9, respectively. The open reading frames were used to design primers for cloning of each respective gene into the *E*. *coli* pET28a expression system adding a C-terminal 6 amino acid histidine tag. Optimal conditions for expression of BbTBP and BbMBF1 were found to be induction at 18°C for 12 h and induction at 30°C for 4 h, before harvesting of cell cultures, respectively. The proteins were purified in a single step by immobilized metal affinity chromatography using Co^2+^-agarose as detailed in the Experimental Procedures section (Fig 1).

### Kinetics of TBP binding to the TATA box and formation of a ternary MBF1-TBP-DNA complex

**Fig 2:**
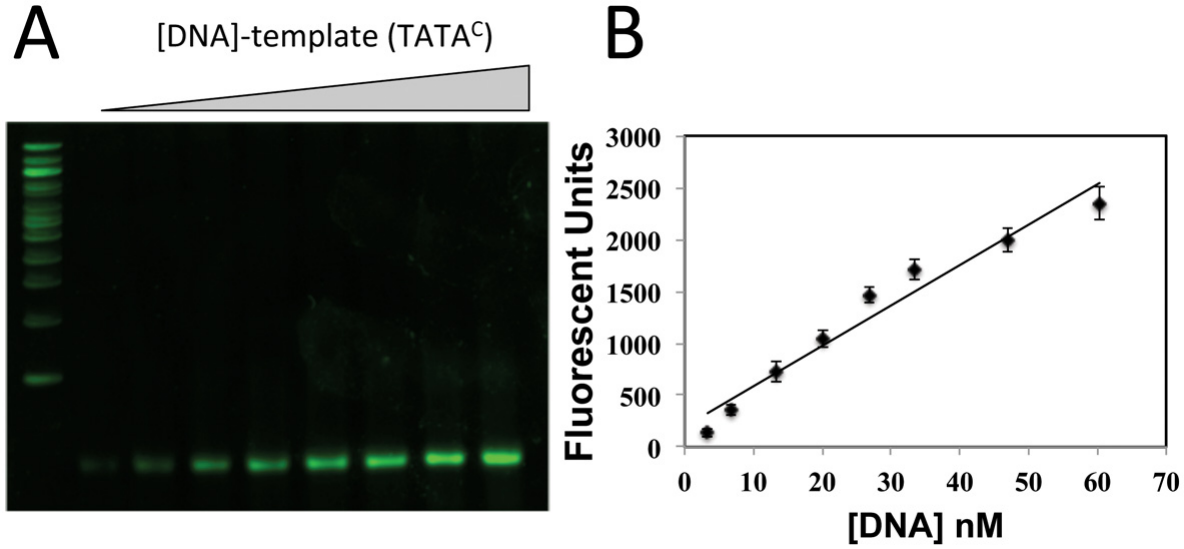
Sensitivity of nucleic acid stain. (A) Increasing concentrations of TATA^C^-oligonucleotide (0, 3.35, 6.7, 13.4, 20.1, 26.8, 33.5, 46.9, and 60.3 nM) were resolved on 6% non-denaturing SDS-PAGE and analyzed as detailed in the Experimental procedures section. (B) Quantification of fluorescent signal from panel (A). Linear fit: y = 38.94x + 196.3; R² = 0.95782.

**Fig 3:**
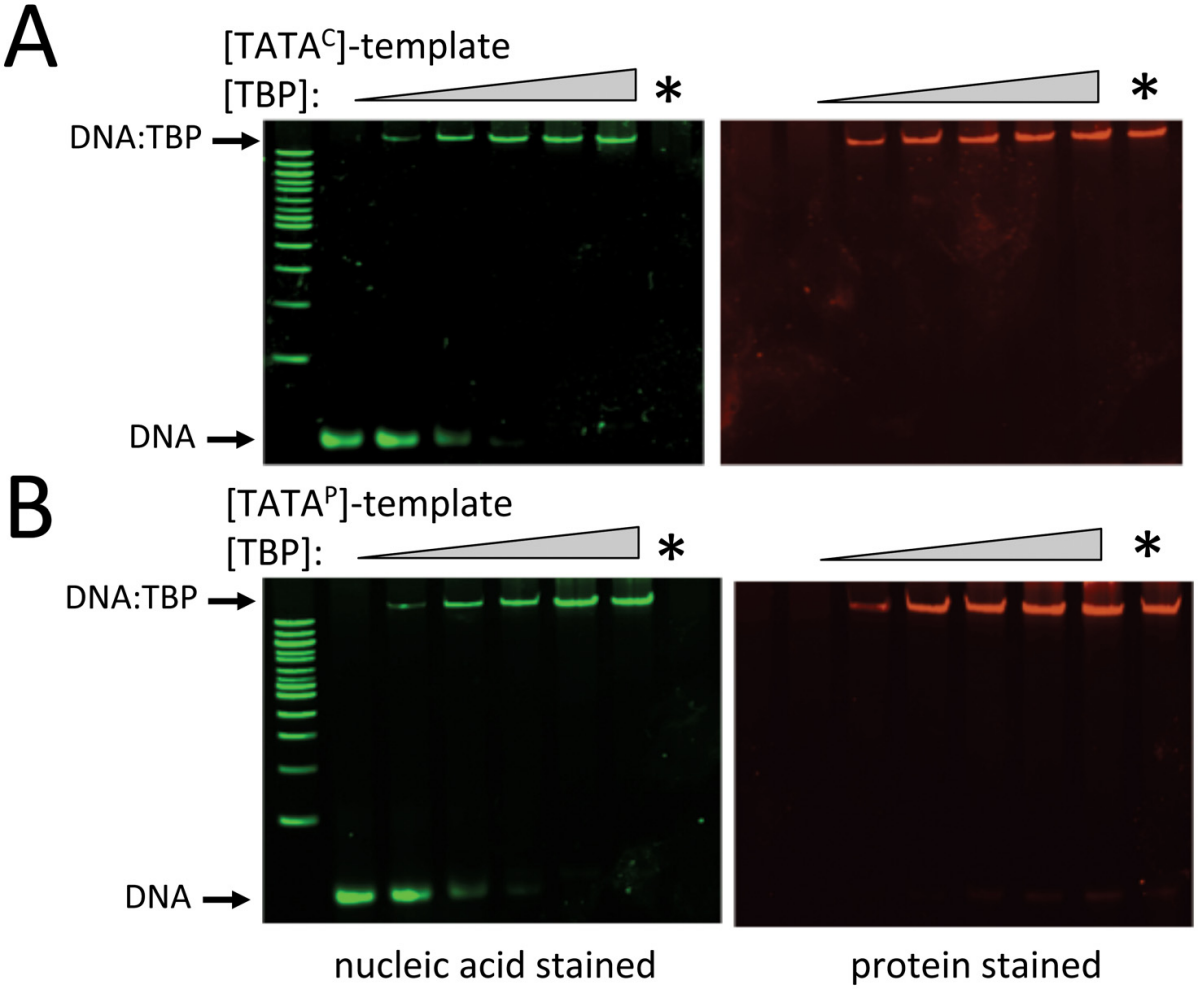
EMSA analysis of BbTBP binding to TATA-containing oligonucleotides. (A) Binding of purified BbTBP to TATA^C^-template. Increasing concentrations of BbTBP (0, 280, 560, 1120, 1680, and 1960 nM) were incubated with TATA^C^-template (16.8 nM) and analyzed on 6% non-denaturing PAGE as detailed in the Methods section. Lanes labeled with an asterisk; 1680 nM BbTBP with no template. Left panel, gel visualized with nucleic acid strain (green), right panel, gel visualized with protein stain (red). (B) Binding of purified BbTBP to TATA^P^-template. Increasing concentrations of BbTBP (0, 280, 560, 1120, 1680, and 1960 nM) were incubated with TATA^C^-template (18.3 nM) and analyzed on 6% non-denaturing PAGE as detailed in the Methods section. Lanes labeled with an asterisk; 1680 nM BbTBP with no template. Left panel, gel visualized with nucleic acid strain (green), right panel, gel visualized with protein stain (red).

**Fig 4:**
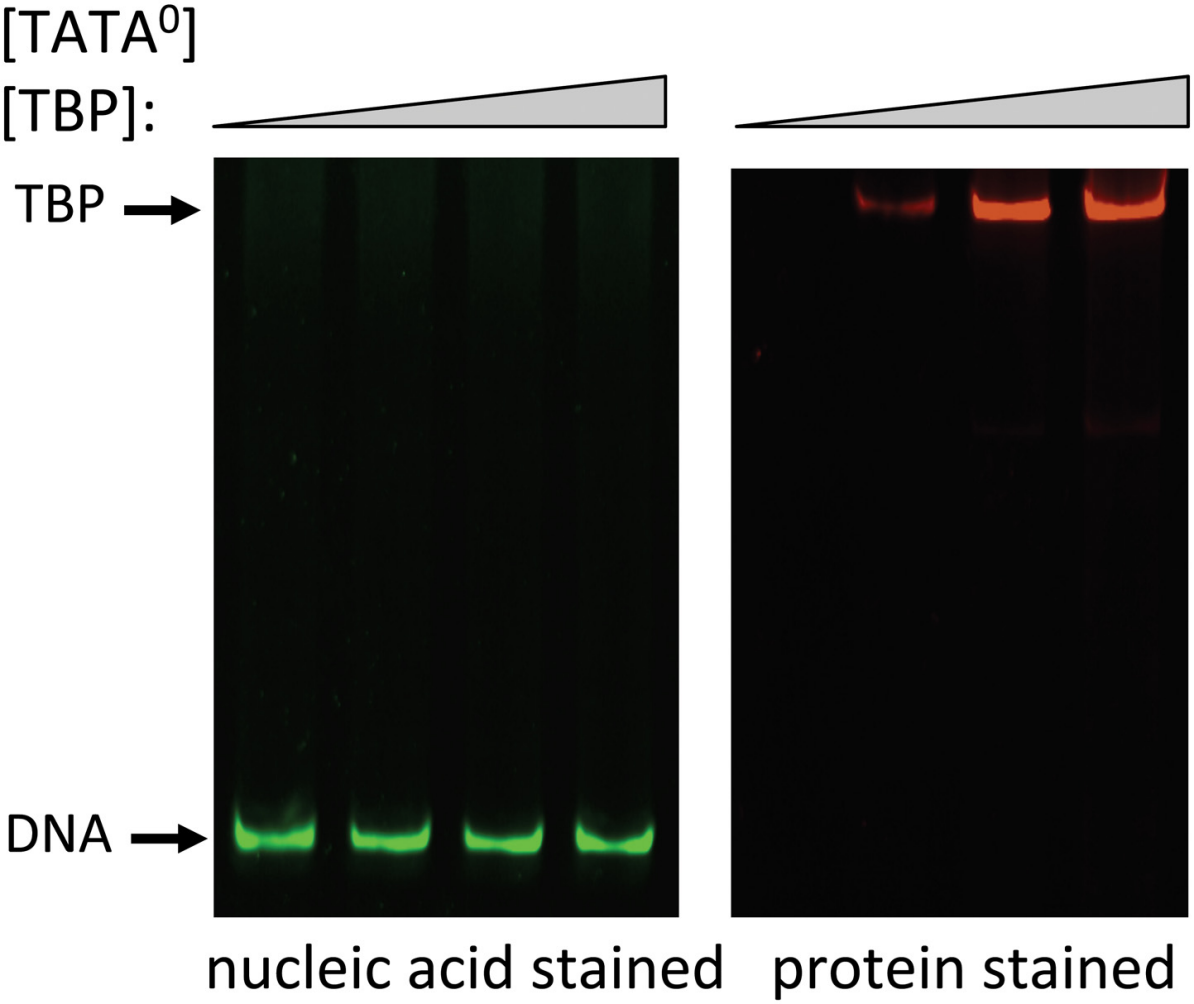
Control EMSA experiments showing no gel shift in reaction mixtures containing purified BbTBP and a TATA-less oligonucleotide. Reaction mixtures containing TATA^0^ template (16 nM) with increasing concentrations (0, 280, 1120, and 1960 nM) of BbTBP, were resolved on 6% non-denaturing SDS-PAGE and analyzed as detailed in the Experimental procedures section. Left panel visualized with nucleic acid stain (green), right panel, protein stain (red).

**Fig 5:**
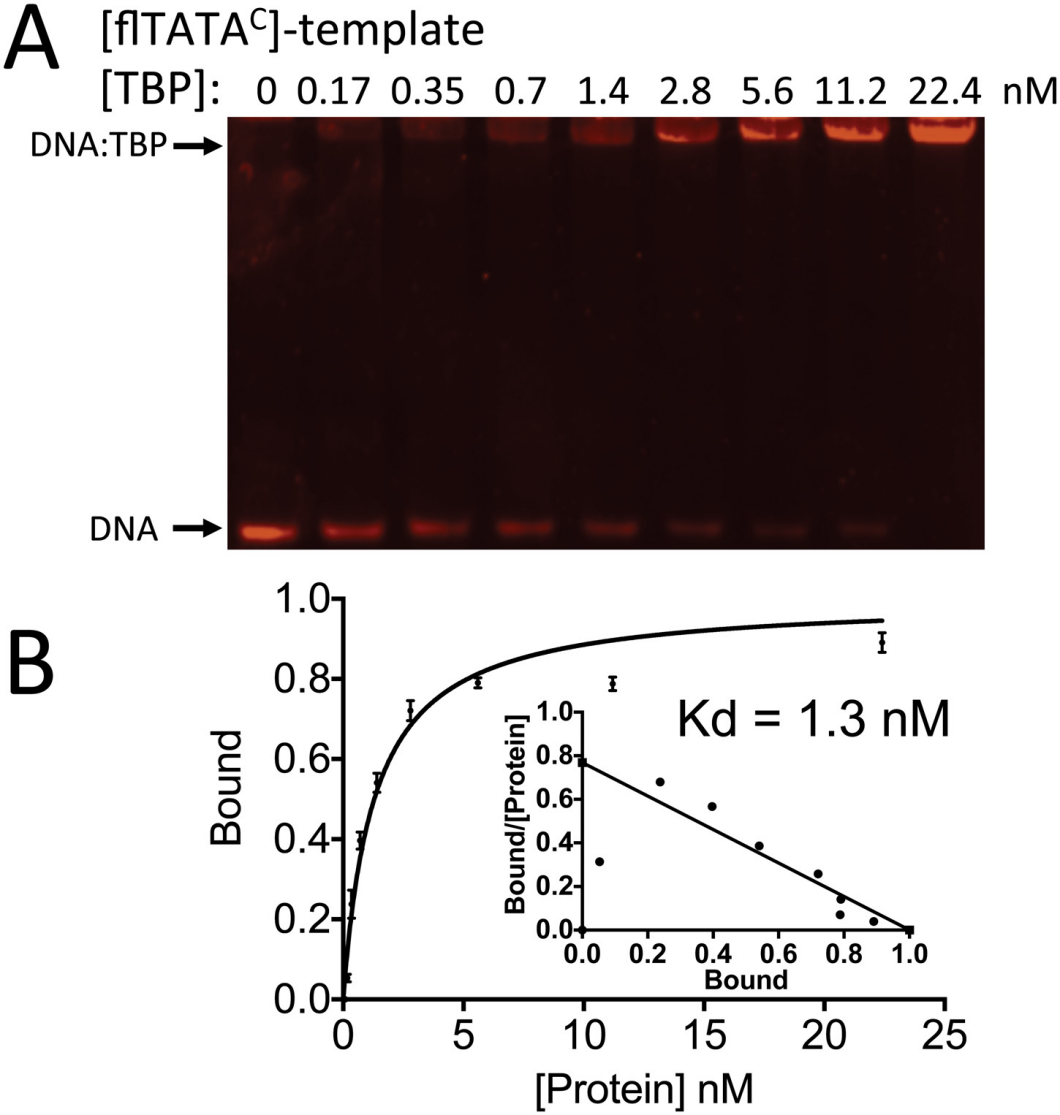
EMSA analysis of BbTBP binding to flTATA^C^ oligonucleotide. (A) Gel shift on 6% non-denaturing PAGE using 0.1 nM fluorescent labeled TATA^C^-oligonucleotide and indicated concentrations of purified BbTBP (0–22.4 nM). (B) Quantification and analysis of BbTBP binding to flTATA^C^ oligonucleotide. Data fit to Y = B_max_ X/(Kd + X), resulting in a K_d_ = 1.3 ± 0.16 nM, B_max_ = 1, R^2^ = 0.97.

Preliminary sets of experiments were performed examining the binding of BbTBP to oligonucleotides derived from *B*. *bassiana* promoter sequences and well as one in which the TATA-sequence was removed. A 40 bp sequence containing two potential TATA motifs was derived from the *B*. *bassiana* subtilisin-like protease promoter (gene locus BBA_04617). The first element (cggcATATgt), however poorly conformed to the canonical TATAAAAAA/G consensus, whereas the second elements (**TATA**AAGAG) was a better match. The second sequence used (40 bp), derived from the chitinase (GenBank: AY145440.1) promoter contained a single element that only poorly conformed to the consensus (att**TATA**tcttc). The constructs were termed TATA^P^ and TATA^C^, respectively, and a control 35 bp sequence lacking the TATA sequence in TATA^C^, termed TATA^0^ was also constructed. Two electrophoretic gel mobility shift (EMSA) assay protocols were used to examine TBP binding to DNA. The first involved nucleotide and protein staining of the gels in which the target DNA template was not labeled, followed by densitometric quantification of the visualized bands [27]. Nucleic acids were stained using SYBR Green, and a standard curve of staining of the TATA^P^ template indicated a linear sensitivity between 3–70 nM (Fig 2). Gel shifts were observed in reactions containing BbTBP and TATA^P^ or TATA^C^, but not when the protein was incubated with the TATA^0^, template (Figs 3 and 4). Although these data provided qualitative confirmation of BbTBP binding to TATA-sequences, quantitative analyses and curve fitting (for K_d_ determination) of these data were not possible due to the high concentrations of protein and template needed for detection using this method. In this method, for [BbTBP] >> K_d_, the binding curve saturates at ~ 1:1 resulting in a curve profile for which accurate fitting of the data was problematic, and the best approximation that could be calulated was a K_d_ < 500 nM. In order to increase the sensitivity needed for accurate affinity constant determination, a fluorescent-tagged version of the TATA^C^ template (flTATA^C^) was synthesized, enabling use of protein concentrations ranging from 0.2–22 nM. Quantification of the binding kinetics resulted in a K_d_ = 1.3 nM for BbTBP binding to the TATA-sequence (Fig 5). Competition experiments were performed to confirm the specificity of binding. Addition of unlabeled TATA^C^ or TATA^P^ templates were effective in competing off BbTBP from the flTATA^C^ template, whereas no competition was seen upon addition of the TATA^0^ template (data not shown).

**Fig 6:**
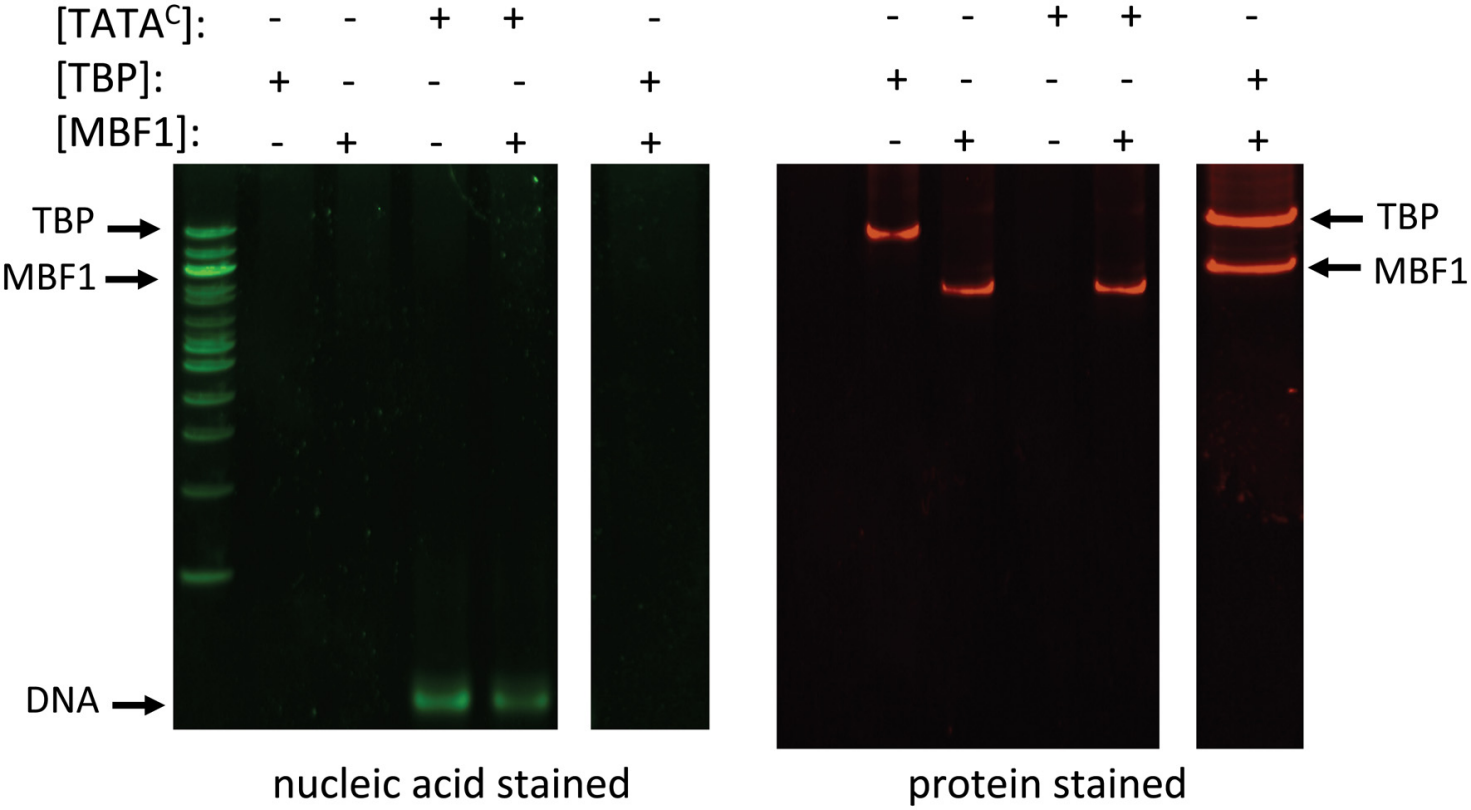
Control EMSA experiments containing indicated proteins in reaction mixtures. Concentrations of TATA^C^ = 18.3 nM, TBP = 1129 nM, and MBF1 = 855 nM. Left panels visualized with nucleic acid stain (green), right panels, protein stain (red).

**Fig 7:**
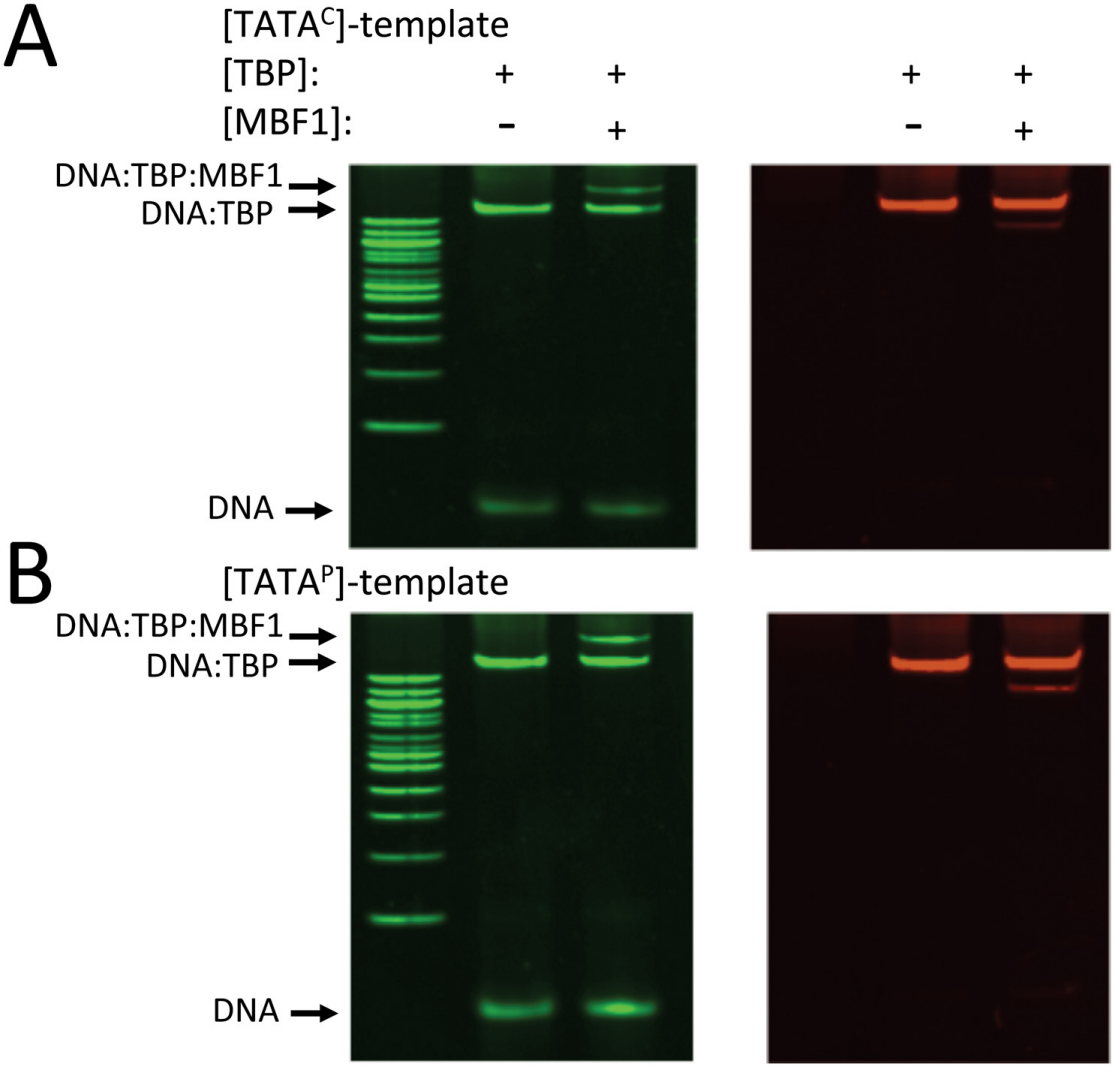
EMSA analysis of BbMBF1-TBP-TATA-oligo complex formation. (A) Interaction of BbTBP and BbMBF1 to TATA^C^-template. BbTBP (1129 nM) was incubated with TATA^C^-template (16.8 nM) with and without BbMBF1 (855 nM) and analyzed on 6% non-denaturing PAGE as detailed in the Methods section. Left panel, gel visualized with nucleic acid strain (green), right panel, gel visualized with protein stain (red). (B) Interaction of BbTBP and BbMBF1 to TATA^P^-template. BbTBP (1129 nM) was incubated with TATA^C^-template (18.3 nM) with and without BbMBF1 (855 nM) and analyzed on 6% non-denaturing PAGE as detailed in the Methods section. Left panel, gel visualized with nucleic acid strain (green), right panel, gel visualized with protein stain (red).

In order to test whether BbMBF1 could bind to the DNA templates directly, a series of EMSA experiments were performed using purified BbMBF1 and the oligonucleotide templates. No gel shift was seen in reactions containing BbMBF1 and any of the template sequences, i.e. TATA^0^, TATA^C^, or TATA^P^ (Fig 6). However, super gel-shifts were seen in reaction mixtures containing the purified proteins, BbMBF1 and BbTBP, and the oligonucleotide targets TATA^C^ (Fig 7A) and TATA^P^ (Fig 7B). No shifts were seen when these proteins were incubated in the presence of the TATA^0^ template (data not shown). Reactions containing the individual proteins, and their combination in the absence of template indicated that BbTBP and BbMBF1 did not appear to form a stable complex (in the absence of DNA template) under the conditions of the (non-denaturing) polyacrylamide gel electrophoresis (Fig 6).

## Discussion

*B*. *bassiana* is an insect pathogenic fungus that is ubiquitously distributed in almost all ecosystems and is currently being exploited as a biological control agents of a wide range of insect pests, where molecular manipulation hold promise for increasing the effectiveness of the fungus in various applications[15,28–30]. The fungus and their insect hosts are engaged in a co-evolutionary arms race, in which, for certain targets, the fungus is winning whereas for others the insect remains ahead [31]. Little is known, however, concerning the underlying transcriptional machinery involved in mediating *B*. *bassiana* gene expression. TBP and MBFs are highly conserved proteins involved in transcription found in all eukaryotes and archaea examined to date, but are absent in prokaryotes. TBP nucleate assembly and position of RNA polymerase pre-initiation complexes. Amongst DNA binding proteins, TBP is unique as it binding in the minor groove of target sequences, resulting in significant bending of the DNA, although important differences between eukaryotic and archeal TBPs in mediating DNA bending has been noted [32–33]. Furthermore, structural analyses have revealed that the saddle-shaped archaea and eukaryotic TBPs can be in opposite orientations when bound to TATA-sequences when driving transcription in the same direction [34,35]. MBF proteins have been grouped into three families; group I that contains the archaea proteins, group II contains the animal, protist, select fungal, and plant proteins, and group III with yeast and filamentous fungal proteins [7,8,36]. The *B*. *bassiana* MBF1 belongs to the group III family, within which the filamentous fungal MBF lineages separate from the yeast proteins in a phylogenetic analysis.

Both *B*. *bassiana* TBP and MBF1 were purified from a heterologous *E*. *coli* expression system. Although both proteins were expressed from the same expression vector, the conditions and yield of each protein was different. BbMBF1 was induced to high levels and could be readily purified from the recombinant host. In contrast, BbTBP1 expression was low and significantly less protein could be purified/L of growth culture. As the expression vectors were constructed from cDNA clones, this could be due to differences in transcriptional and/or translational efficiencies of the constructs. In addition, BbTBP was prone to the formation of aggregates/precipitation. Purified BbTBP was shown to bind to TATA-containing oligonucleotide sequences but not to TATA-less sequence by EMSA analysis. Initial experiments using a dual dye, i.e. nuclei acid and protein staining protocol [27], while successful in qualitatively showing TBP-DNA binding, required protein and template concentrations that were >> K_d_ values, resulting in data that could not be accurately quantitatively analyzed. Use of a fluorescently labeled template, however, resulted in experiments within the appropriate range for analysis, yielding a K_d_ = 1.3 nM for BbTBP binding to the TATA-sequence. This number is similar to that reported for yeast TBP, i.e. 4–5 nM [37] and from 0.44 to 5 nM for TATA-binding protein interactions to variously modified TATA boxes [38,39]. The oligonucleotide sequences used were derived from the *B*. *bassiana* chitinase and protease genes [26], enzymes critical for targeting of insects hosts by the fungus [40,41]. Indeed, biotechnological application of a fusion chitinase-protease protein has shown promise as a means of increasing the ability of the fungus to target insect hosts [42,43]. Both enzymes are considered inducible [44–46], and consistent with the presence of TATA-sequences in the promoter regions of inducible genes, putative TATA-motifs were identified in the promoter regions of both genes. The protease promoter sequence contained two TATA-sequences, one of which conformed poorly to the context consensus sequence, whereas the other motif was a better match. The chitinase TATA-sequence also did not conform to the canonical TATA-sequence consensus. Our data indicate that both template sequences were capable to mediating TBP binding, and that TBP affinity to the motif found in the chitinase promoter was comparable to that seen in other systems. These data are consistent with a recent survey of TBP binding sites in which chromatin immunoprecipitation (ChIP) was used to identify novel human genome TBP binding sites. Many such sites were located in introns or lacked gene/mRNA annotation. However, the authors report that these sites could still direct transcription, suggesting widespread targeting of TBP and associated complexes to non-canonical sites [47].

Despite MBF proteins having one (putative) DNA-binding helix-turn-helix (HTH) domain [48], these proteins do not bind DNA directly, a finding confirmed by our results. *B*. *bassiana* MBF1 was unable to bind to oligonucleotides irrespective of the presence of a TATA-motif, whereas BbTBP was dependent upon the presence of a TATA-motif in the target DNA. A super shift in EMSA experiments was noted in reactions containing TATA-motif DNA targets, BbTBP, and BbMBF1, thus providing evidence for ternary complex formation between these partners. Our data are consistent with a model in which TBP bridges MBF1 with target DNA sequences. As MBF1 is supposed to then bridge transcription factors to the (TBP-containing) initiation complex future work identifying the partners of BbMBF1 is warranted. Only a handful of transcription factors have been characterized in *B*. *bassiana*. The carbon catabolite repressor transcription factor, *BbcreA* has been shown to be important for nutrient assimilation, development, differentiation, temperature sensitivity, and virulence [49], and the stress response transcription factor *Bbmsn2*, has been linked to production of secondary metabolites, pH dependent growth, and stress response [50]. Intriguingly, putative binding sites for CreA and Msn2 have been identified in the promoter sequences of the chitinase and protease genes, however, whether there are any links between these transcription factors and MBF1 remains to be determined.
